# Breast Cancer in Young Women: Status Quo and Advanced Disease Management by a Predictive, Preventive, and Personalized Approach

**DOI:** 10.3390/cancers11111791

**Published:** 2019-11-14

**Authors:** Erik Kudela, Marek Samec, Peter Kubatka, Marcela Nachajova, Zuzana Laucekova, Alena Liskova, Karol Dokus, Kamil Biringer, Denisa Simova, Eva Gabonova, Zuzana Dankova, Kristina Biskupska Bodova, Pavol Zubor, Daniela Trog

**Affiliations:** 1 Department of Obstetrics & Gynecology, Jessenius Faculty of Medicine, Comenius University in Bratislava, Martin University Hospital, 03659 Martin, Slovakia; marek.samec@uniba.sk (M.S.); marcela.nachajova@uniba.sk (M.N.); zuzana_laucekova@yahoo.com (Z.L.); alenka.liskova@gmail.com (A.L.); kamil.biringer@uniba.sk (K.B.); denisa.simova@gmail.com (D.S.); kristina.biskupska@uniba.sk (K.B.B.); pavol.zubor@uniba.sk (P.Z.); 2 Division of Oncology, Biomedical Center Martin, Jessenius Faculty of Medicine, Comenius University in Bratislava, 03659 Martin, Slovakia; peter.kubatka@uniba.sk (P.K.); zuzana.dankova@uniba.sk (Z.D.); 3 Department of Medical Biology, Jessenius Faculty of Medicine, Comenius University in Bratislava, 03659 Martin, Slovakia; 4 2nd Department of Gynecology and Obstetrics, Hospital of F.D. Roosevelt, Slovak Medical University, 97401 Banska Bystrica, Slovakia; dokusk@yahoo.com; 5 Clinic of Surgery and Transplant Center, Jessenius Faculty of Medicine in Martin, Comenius University in Bratislava, 03659 Martin, Slovakia; egabonova@gmail.com; 6 University Clinic of Radiooncology, Westfälische Wilhelms Universität Münster, 48149 Münster, Germany; Daniela.Trog@ukmuenster.de

**Keywords:** breast cancer, young population, premenopausal, risk factors, screening, individualized patient profile, epidemic, prevalence, paradigm change, predictive preventive and personalized medicine, etiology, body shape, Flammer syndrome, systemic hypoxia, interpretation, patient stratification, innovative concepts, phenotyping, genotyping

## Abstract

Why does healthcare of breast cancer (BC) patients, especially in a young population, matter and why are innovative strategies by predictive, preventive, and personalized medicine (PPPM) strongly recommended to replace current reactive medical approach in BC management? Permanent increase in annual numbers of new BC cases with particularly quick growth of premenopausal BC patients, an absence of clearly described risk factors for those patients, as well as established screening tools and programs represent important reasons to focus on BC in young women. Moreover, "young" BC cases are frequently "asymptomatic", difficult to diagnose, and to treat effectively on time. The objective of this article is to update the knowledge on BC in young females, its unique molecular signature, newest concepts in diagnostics and therapy, and to highlight the concepts of predictive, preventive, and personalized medicine with a well-acknowledged potential to advance the overall disease management.

## 1. Introduction

Although the mortality rates of BC have been stabilized, the annual numbers of new cases are permanently increasing [1]. Even though the majority of BC patients are postmenopausal women, there is a quick growth of the premenopausal BC cohort. Consequently, the ratio between post- and premenopausal BC shifts, which leads to younger BC age-profile than in the previous century [1,2]. Whereas, postmenopausal BC is frequently linked to well-detectable risk factors such as overweight, obesity, and diabetes type 2, the heterogeneous phenotype and genotype of premenopausal BC hinders risk assessment, early diagnosis, cost-effective treatment, and long-term disease-free outcomes [3]. To this end, triple-negative BC (TNBC), a subtype with the poorest outcomes, is more typical for premenopausal women [4]. Moreover, “young” BC is frequently “asymptomatic” [5]. Additionally, systemic hypoxic effects have been demonstrated to contribute to particularly aggressive metastatic disease in BC patients [6]. Furthermore, the relevance of the Flammer syndrome (FS) phenotype (which is typical rather for young females) for BC development and progression specifically in young women has been demonstrated in recently published literature sources, such as *Flammer Syndrome—From Phenotype to Associated Pathologies, Prediction, Prevention and Personalization* and gets analyzed in the below-provided sections [7]. In contrast to an effective golden standard of mammography screening established for middle-age and elderly, screening programs for BC in young females are still underdeveloped [8]. Moreover, around 250 articles across all areas were awarded by *Springer Nature* a title "groundbreaking scientific findings that could help humanity and protect our planet" in 2018 [9]. An awarded article in the category “Medicine and Public Health” [10] entitled “Pregnancy-associated breast cancer: The risky status and new concepts of predictive medicine”, focused on the pregnancy-related malignancy of the mammary gland, presented innovative concepts for the paradigm shift to the predictive preventive and personalized approach [11]. However, impeded and delayed diagnosis of pregnancy-associated BC (PABC), as the most prevalent type of malignancy during pregnancy, combined with aggressive cancer type, leads to the dramatic decline in overall survival in the PABC group [12,13]. Currently, any population screening program, standardized diagnostic approach, generally accepted factors associated with risk assessment or measures of prevention targeting particularly PABC, have not been established [14].

Accordingly, an objective of this article is to update the knowledge of BC in young females and to highlight the concepts of predictive, preventive, and personalized medicine with a well-acknowledged potential to improve the overall disease management [15].

## 2. Epidemiology

According to Globocan statistics, BC is the second most common malignancy worldwide with 2,088,849 expected new cases in 2018. BC is by far the most common malignant disease in the female population followed by lung, cervical, and thyroid cancer. The worldwide incidence is 46.3/100,000 women with the mortality rate 13.0/100,000 for women [1]. These numbers are much higher in the developed world. Despite the risk for BC in the third decade of life being only 0.04%, it is still the most common cancer in women younger than 35 [16]. There is also emerging evidence of a rising trend of BC in young women at the age of ≤40. The Group for Cancer Epidemiology and Registration in Latin Language Countries (GRELL) study analyzing epidemiologic data from European countries showed the increasing rate by 1.2% annually between 1990–2008 [2].

In Europe, BC represents 26.4% of all female malignancies with 522,513 new cases in 2018. BC remains the most common invasive disease also in young females aged 20–39 with 32,425 new cases (ASR 28.4/100,000), followed by cervical cancer, thyroid cancer, and melanoma. Mortality in this category is low and reached 2883 cases in 2018 (ASR 2.5/100,000) [1]. BC remains the single greatest cause of cancer-related deaths in young adult women; nevertheless, the diagnosis of females younger than 40 years of age is associated with only approximately 5% of all BC. According to the statistics in the UK, BC is extremely rare in the 20–24 age group (ASR 1.5/100,000). However, it is steadily rising with age and its incidence in the group of 34–39-year-old females is 65.1/100,000 [17].

## 3. Risk Factors 

Many risk factors (modifiable/non-modifiable) are included in BC prediction models. However, the proportion of BC cases related to these factors stratified in accordance with the menopausal status is unknown [3,11]. Moreover, BC risk factors are not the same for pre- and postmenopausal women [15]. A very brief summary of common BC risk factors is presented in Table 1.

Generally, obesity as a risk factor in postmenopausal age could be protective in younger women [2,18]. Moreover, multiparity, known as protective factor in postmenopausal females, is a risk factor for women of childbearing age due to the transient increase in risk in the 12 months postpartum. Pregnancy therefore does not increase the risk of BC, but accelerates those cancers in early stages that are present during pregnancy. Other common risk factors connected to early-onset BC include high alcohol intake, smoking, radiation exposure in utero, and family history of BC at a young age [19,20,21]. Furthermore, almost 10% of all BC cases are caused by genetic mutations [22]. A high proportion of BC patients younger than 40 carry a *BRCA1*/*BRCA2* mutation. These mutations significantly affect the overall survival of BC patients. On the other hand, *BRCA* mutations in patients with TNBC is linked to better survival when compared with non-carriers [23]. *TP53* and CHEK2*1100delC mutations are also associated with early-onset BC [24,25]. Surprisingly, TP53 mutations are responsible for almost one fifth of all hereditary malignancies and are also present in 2–6% of BC cases in women younger than 35 [26,27,28,29]. Additionally, BC microenvironment is a focus of multiple studies with a particular interest in FS. Hypoxic environment plays a crucial role in tumor promotion and is associated with strong epi/genetic BC predisposition. FS generally affects young females demonstrating specific molecular patterns including endothelin-1 (ET-1) and activity of metalloproteinases MMP-9, MMP-2 characteristic for BC, and metastatic disease with poor prognosis [15,30]. FS phenotype may act over several years or decades as a strong risk factor for BC with poor prognosis [6]. The FS relevance for BC is described in more details below.

## 4. Pathological Characteristics and Tumor Behavior

As discussed above, BC is a dominant malignant disease generally affecting postmenopausal women older than 40 years. On the other hand, an incidence of the advanced BC in younger women (premenopausal) is increasing. Many studies focusing on age stratification demonstrated that mammary carcinogenesis in younger premenopausal women has a worse prognosis and more unfavorable molecular subtypes in comparison with older postmenopausal patients [31]. Therefore, young age at the time of cancer development is an independent risk factor associated with higher recurrence and mortality even when the more aggressive treatment is applied [32].

### 4.1. Pathology of BC in Adolescent and Young Adult Patients

As was demonstrated by numerous large-scale studies, distinct molecular subtypes contribute to defining the strategy of personalized medicine and thus predict optimal therapy [33]. As mentioned previously, the development of BC in premenopausal age is connected to molecular subtypes and indicates poorer prognosis [34]. Based on molecular differences primarily through gene expression and presence/absence of hormonal receptors on the surface of cell, four clinically relevant BC subtypes have been identified, including Luminal A (ER+, PR+, HER2−, Ki67 low expression), Luminal B (ER+, HER2−, PR−, or Ki67 high expression), TNBC (ER−, PR−, HER2−), and HER2 (HER2 overexpression) [4].

The luminal types represent subtypes with better prognosis and success of a therapeutic intervention. On the other hand, TNBC and HER2 molecular subtypes represent the most aggressive forms of mammary carcinogenesis with a worse prognosis [35]. Both TNBC and HER2 subtypes are over-represented in cohorts of very young women with BC (below 40 years) compared to overall world-wide population, e.g., approximately 26% cases of TNBC are identified in very young women, whilst global frequency of the most aggressive forms constitutes 12% of all diagnosed cases [4]. Recently, numerous studies focus on the retrospective observational analysis of differentiation between molecular subtypes and their abundance in premenopausal women [32,36,37]. In a retrospective study, researchers evaluated molecular subtypes and prognostic factors for survival in a cohort of 662 Mexican women with BC below 40 years. The results showed that younger patients (<the 40s) had a more aggressive presentation including higher grade and higher frequency of Luminal B and TNBC, respectively. Worse 5-year overall survival was connected only with Luminal B subtype (79.1% vs. 85.2%, *p* = 0.03) in comparison to older patients with Luminal B [38]. Further investigation in the field of retrospective analysis of differences between premenopausal and postmenopausal cohorts of Afro-American women focusing on their molecular subtypes revealed higher frequency of tumor features with poor prognosis in younger women when compared to older women. Additionally, worse prognostic signature including the negative status of progesterone (PR) and estrogen receptor (ER), larger size, high grade, and triple-negative subtype of the tumor were determined by multivariate analysis in younger women [39]. An increase in expression of genes involved in the cell processes including cycle, repair of DNA damage, and nucleotide metabolism in young female TNBC patients was currently revealed by gene set enrichment analysis [40].

More recently, Ryu and colleagues [41] evaluated BC mortality in association with molecular subtypes according to age among women younger than 50 years in a nationwide Korean study in which subgroups of patients were analyzed in their 20, 30, and 40 years. Based on the result obtained from this large-scale study conducted on 30,793 patients, women in their 20s were significantly more likely to have cancer of higher stage with higher nuclear grade. Moreover, patients from the 20s group exhibited poor prognosis evaluated by multivariate analysis for overall survival and hazard ratio. Subtypes stratification showed that luminal types are associated with worse prognosis in the youngest women compared to patients in other age groups [41]. Furthermore, in a retrospective population-based study, Tang et al. [42] focused on the evaluation of different characteristics and prognosis of BC among young Chinese women. The study included 1360 patients below 40 years-old and a control group with 3110 cases in the 40–50 age group. A significant lower prevalence of Luminal B subtype was observed in the group of younger patients (< the 40s) compared to the control groups (>40s) (23.5% vs. 17.5%, *p* < 0.01). The prevalence of TNBC was similarly high in the group of patients below 40 years (16.7% vs. 13.4%, *p* < 0.01). Luminal A represented the subtype with a lower prevalence in the younger women when compared to the older cohorts (48.5% vs. 59.2% *p* < 0.01). Additionally, differences in clinical outcomes between luminal subtypes were also demonstrated while Luminal B may predict a negative prognosis for young patients associated with overall worse survival rate [42]. Moreover, results of a retrospective observational study evaluating pathological features and prognosis in the Chinese-Han population, including 94 patients aged <25 years, revealed that HER2 positive and TNBC subtypes are defined as negative predictions signatures associated with shorter disease-free survival (DFS). Further, they demonstrated that ER status may be classified as independent prognostic factors for young women with BC [43]. In 2017, Sharma and Singh [44] published a retrospective analysis including 415 patients divided into two groups above 40 years and less than 40 years of age, respectively. The main aim of the study was to evaluate the impact of age on prognosis between younger and older cohorts of patients. The results demonstrated a modest lower prevalence of PR positivity (38.54% vs. 44.76%, *p* = 0.001) and ER positivity (39.84% vs. 55.23%, *p* = 0.005). Interestingly, the triple-negative subtype of BC was in higher prevalence (46.61% vs. 32.21%) in the <40 years group. Consequently, differences in the biological behavior of BC in younger women were associated with poor clinical outcomes and worse prognosis connected with aggressive forms of tumor [44].

In summary, numerous previously mentioned studies demonstrated cross-link between very young age and prognosis of BC. Despite indisputable evidence in differences in the prevalence of the molecular subtypes and therapy approaches in cohorts of very young women compared to older patients, specific insights into biology, distribution, and selection of the most appropriate treatment for women with BC younger than 40 years old are still rare [4,45].

### 4.2. Molecular Signatures of Early-Onset BC

Histopathological, molecular, genetic, and genomic studies have demonstrated that young women with BC show an increased rate of more aggressive subtypes with an overall worse prognosis, increased genetic susceptibility, differential tumor gene expression, specific genomic signatures, as well as alternations in epigenetic events including miRNA expression in comparison with postmenopausal women with BC [46]. Advances in novel technologies such as next-generation sequencing (NGS) and gene microarray have resulted in enormous progression and deluge sequence data about gene polymorphisms or determination of patterns of gene expression implicated in various pathological states [47,48,49,50]. Rummel et al. [50] enrolled female patients within the Clinical Breast Cancer Project between 2001 and 2015 that were diagnosed with invasive BC before the age of 40. The mentioned group of very young women consisted of seven percent (141/1980) of all patients and 44 of them were classified with a family history of BC. The sequencing analysis of genomic DNA was performed in 118 patients. Pathogenic mutations were found in 27 individuals: *BRCA2* (*n* = 12), *BRCA1* (*n* = 10), *TP53* (*n* = 1), and *CHEK2* (*n* = 4). In addition, pathogenic mutations in *APC* (*n* = 1) and *MUTYH* (*n* = 2) genes were also observed. The authors concluded that pathologic changes in high- and moderate-risk BC genes were observed in 23% of very young women (< 40). Moreover, an additional 3% of individuals demonstrated pathogenic mutations in predisposition genes for colon cancer. Variants of uncertain significance were revealed in 14% of young patients in genes like *BRCA2*, *CDH1*, *CHEK2*, *PALB2*, and *ATM* [50].

Moreover, Colak et al. [51] investigated the transcriptomic profile and network evaluations of BC in patients from the Middle East to recognize age-specific molecular signatures of this malignancy. In addition, authors evaluated molecular changes linked with the progression of carcinogenesis in young women using the comparative genomics approach coupled with copy number variants related to BC from other studies. Authors chose five distinctly deregulated genes (*SEPP1*, *ESR1*, *IL1RN*, *SCD*, and *TIAM1)* in very young (≤35 years) and/or young (≤5 years) patients that were compared with older cohorts. A significant correlation (Pearson’s r > 0.76) was found between the real-time RT-PCR and microarray data. The mentioned correlation was higher when the older group (>45 years) was compared with the group of very young women (≤35 years) (r = 0.99) vs. comparison of the cohort of young women (35–45 years) with the group of very young women (r = 0.77). Moreover, they found 63 genes typical for BC in young women that pointed to genomic changes different from two age groups of older patients. The network evaluations demonstrated potential important regulatory functions of PI3K/Akt, Myc, NF-κB, and IL-1 in the molecular features of BC in young patients. Comparative cross-species genomics evaluations of BC progression from pre-invasive DCIS to IDC detected 16 markers with attendant genomic changes, i.e., *UBE2C*, *CCNB2*, *CEP55*, *TOP2A*, *BIRC5*, *TPX2*, *SHCBP1*, *KIAA0101*, *PTTG1*, *UBE2T*, *DEPDC1*, *NUSAP1*, *CCNB1*, *HELLS*, *KIF4A*, and *RRM2* that may be included in BC invasion and progression in young women [51].

Another study conducted on 47 Korean very young ER+ BC women (<35 years) (KYBR) focused on the molecular characteristic based on the assessment of mutations and copy number variants as well as on the analysis of expression profiling. The data were compared with the dataset of The Cancer Genome Atlas (TCGA) including young BC patients with ER+ subtype. *TP53*, *GATA3*, and *PIK3A* genes possessed most recurrent somatic mutations. APOBEC-associated mutation signature of KYBR occurred at a higher frequency when compared with TCGA. Considering the molecular characteristics, integrative profiling created 3 subgroups of patients. Specifically, group A included A-like subtype and IGF1R signal deregulation, and group B and C luminal B women characterized by chromosomal instability and enrichment for APOBEC3A/B deletions, respectively. Furthermore, group B patients included 11q13 (*CCND1*) amplification and activation of the ubiquitin-mediated proteolysis pathway. Moreover, 17q12 (*ERBB2*) amplification and lower ER and PR expressions were observed in group C, which was also determined by immune activation and lower EMT level when compared with group B [52]. The higher incidence of basal-like cancers in very young women may be associated with an increase in the expression of *BRCA1* mutations, luminal progenitors, and c-kit in younger patients [53]. RANKL pathway also appeared as a PgR-regulated gene involved in the expansion of mammary stem cells, an increase in their proliferation, and a decrease of apoptosis [54].

Novel multi-omics approaches identified the difference in molecular signature between cohorts of younger and older women with TNBC [40]. In the study of Ma et al. [40], authors focused on differences between somatic mutations in women with TNBC and DNA damage features. The results showed a parallelism between different age groups and mutation profiles, but on the other hand, distinct generation mechanisms implicated in homologous recombination deficiency were presented. Higher frequency of the germline mutations in *BRCA1* was detected in cohorts of young women with TNBC, while mutation rates in *BRCA2* were relatively identical among groups with TNBC. Interestingly, misbalance in the frequency of mutation in the *BRCA2* gene (copy number loss of Chr13q13) was detected in groups of younger women primarily (*p* < 0.05). Moreover, the evidence suggested the higher frequency of mutation such as copy number loss at chromosome 15 (Chr15q13. with FAN1 in the ’peak’) and changes (amplification) in gene copy number (Chr1p34, with KDM4A in the ’peak’) in patients with early-onset BC. These two findings significantly influenced *FAN1* and *KDM4A* gene expressions and both events were amended with genomic-based homologous recombination deficiency indexes [40].

Specific genetic variants have been characterized as either predictive or prognostic biomarkers. BRCA1/BRCA2/Rad51 complex is suggested to be one of the promising prognostic biomarkers, especially in women with BC younger than 40 years. Moreover, this complex plays a key role in the repair activity of the signaling pathway of homologous recombination [55].

Söderlund et al. [56] found that low expression of BRCA1/BRCA2/Rad51 complex is a marker of poor prognosis of early BC. On the contrary, low expressed complex responded well to radiotherapy compared to women with a high expression that demonstrated few local recurrences and no additional benefit from radiotherapy [56]. Studies demonstrated that inflammation biomarkers may also be clinically valuable in high-risk women for early BC [57]. In this regard, the TNF-308G>A polymorphism has been demonstrated as a marker with significant prognostic value for early BC survival [58]. Compared to homozygous genotype carriers, heterozygous individuals demonstrated a significant decrease in progression-free survival, metastasis-free survival, and overall survival. On the other hand, IL10 -1082A > G, -819 T > C, -592A > C polymorphisms and phased haplotypes have not shown a prognostic value for early BC in the same study. Zhang et al. [59] described that the decreased risk of BC (OR = 0.62; 95% CI 0.41–0.94) associated with regular Nonsteroidal anti-inflammatory drugs (NSAID) administration was restricted to younger (premenopausal) patients only [59].

Global miRNA expression profile reveals novel predictive and prognostic biomarkers in different age groups including cohorts of very young women below 40 years of age. Several experimental studies suggested different amplitudes in expression patterns of various miRNAs between younger and older women with BC [46]. Recently, Peña-Chilet et al. [60] focused on an association between early-onset BC in young individuals and clinical-pathological features of the tumor. The authors performed a comprehensive study aimed to differentiate miRNAs expression in the cohort of very young women (≤35) compared to older patients. The results suggested alterations in levels of several miRNAs, including higher expression of miR-1228, miR-3196, miR-1275, and miR-1207 and lower expression of miR-92b and miR-139 that are related to modulation of cellular mechanisms, such as invasion or proliferation [60]. In another study, Tsai and colleagues [61] evaluated specific miRNA expression patterns in different age groups and pathological subtypes of BC in Taiwan. Experimental data revealed an increased level of miR-183, miR-182, and miR-96 as well as decreased level of miR-320, miR-10a, miR-130, miR-127-3p, miR-143, miR-10b, miR-125b, and miR-195, respectively. All the previously mentioned miRNAs were found to be specifically deregulated in very young women (≤35) and may participate in the tumor initiation in early age [61]. Furthermore, a retrospective study on 258 very young women (≤35) that were diagnosed with BC demonstrated changes in miRNAs expression. Disclosure of candidate miRNAs was performed using microarray technology that validates the correlation between levels of differentially expressed miR-30c and poor-prognosis in these women. The downregulation of miR-30c was associated with more aggressive forms of cancer and the development of lymph node metastasis [62].

Most recent data pointed to a significant role of receptor activator of nuclear factor κB (RANK)/RANK ligand (RANKL) signaling in BC development. In this regard, RANK/RANKL inhibition suppressed progestin-induced carcinogenesis and mammary stem cell component in preclinical models of BC [63]. Therefore, targeting RANK/RANKL signaling may represent a reasonable non-surgical prevention choice for carriers of BRCA mutation including very young women [64,65]. The molecular signatures associated with early-onset BC are reviewed in Table 2.

## 5. Screening and Diagnostics 

The role of BC detection in healthy women with average risk is not established in routine screening by any imaging method. However, the interplay of various diagnostic factors, such as lack of routine screening, the decline in the mammography sensitivity, as well as underestimation of mammary pathologies are associated with a higher frequency of advanced stage of BC in young women. Each abnormal finding on breast self-examination should be carefully tested by the triple test—physical breast examination, imaging test, and cytopathologic/histopathologic analysis.

Ultrasound alone is not an acceptable or validated screening tool in young women but it is the preferred imaging method in pregnant and symptomatic women under 30 [66]. If there is a need for further testing, digital mammography is superior to film mammography in dense breasts and is more sensitive in young patients [67]. Novel mammographic techniques such as tomosynthesis may be promising especially in unmasking malignancies in dense breasts in young women. The standard of breast imaging characterized by improved screening and diagnostic outcomes is attributed to digital breast tomosynthesis (DBT) which was approved by the Food and Drug Administration (FDA) in 2011 [68]. As was demonstrated by prospective European studies, the addition of DBT led to an increase in cancer detection rate (CDR) from 27% to 91% [69,70]. Moreover, assistant diagnostic markers such as DBT-determined lymph node size, as well as calcification score, could be used also to determine BC molecular subtypes [71]. A comparison of TOMosynthesis with digital MammographY in the UK NHS Breast Screening Programme (TOMMY) trial showed the superior value of DBT for women with a breast density of 50% or more with sensitivity 93% and specificity 70% compared to 2D mammography (86% sensitivity and 57% specificity) [72]. Despite the progress in the clinical availability and utilization of tomosynthesis, no specific evidence focusing on its use in young women is accessible; therefore, the use and indications are identical with those in other age groups [73].

When compared to mammography, magnetic resonance imaging (MRI) is not affected by breast density and is more sensitive in tumor size assessment and evaluation of multifocal lesions. On the other hand, evidence of effective screening using magnetic resonance in the setting of young women without a genetic BC predisposition is missing. Importantly, annual mammographic screening is recommended by the American College of Radiology (ACR) for women of average risk at age 40. However, the mammography screening of women at higher risk should start earlier. Moreover, they may profit from screening modalities such as MRI. Supplemental screening with contrast-enhanced breast MRI is recommended for females with a lifetime BC risk of at least 20% or a history of chest radiation therapy at a young age. Dense tissue is another reason for the recommendation of breast MRI [74].

Exponential growth in the field of imaging methods contributed to the development of radiomics representing a comprehensive characterization of tumors based on various quantitative features acquired from digital images. Actually, radiomics may participate with the use of imaging biomarkers in BC diagnosis as well as in the prediction of treatment responses and recurrence risk [75]. This complex analysis and possibility of an individualized approach can be applied also in women under the age of 40. Moreover, whole-tumor radiomics on MR multiparametric images provide a non-invasive analytical approach for BC subtype classification and TNBC identification. This novel approach is named radiogenomics [76].

Imaging methods are nowadays a gold standard in BC diagnosis. The disadvantages of mammography screening include higher false-positive cases, excessive biopsies, and irradiation linked to excessive mammography (MMG) use. No evidence has demonstrated an association between higher frequency of mammography screening or screening with other modalities and the decrease in the risk of BC in women at low or intermediate lifetime risk of the disease development. Moreover, genetic-associated risk stratification seems to be more profitable in comparison with age-stratified approaches. Enhancement of risk prediction could depend on the combination of mammography screening, proteomics, genomics, and classical risk factors [77].

## 6. Therapy

Young women should not undergo aggressive therapeutic modalities only because of their age. The choice of treatment should be based on biological characteristics of the tumor, stage, grade, genetic status, patient´s co-morbidities, and preferences [73]. On the contrary, young women are often diagnosed in an advanced BC stage [78]. Therefore, general surgical treatment of young patients with BC should be the same as in older patients. The first option should be breast-conserving surgery as it provides the same overall survival than mastectomy. There is no evidence of better overall survival after bilateral mastectomy in young women without genetic mutations including *BRCA1* and *BRCA2* [73]. Studies have investigated trends in treatment choice for stage 1 and 2 BC. Increasing rates of conservative treatment compared to mastectomy were observed after 1990. However, the rising trend in mastectomy was published in recent articles since 2000 [79]. Lazow et al. [80] showed that the majority of patients underwent unilateral or bilateral mastectomy (57.2%) rather than Breast conserving therapy (BCT) (42.8%). Mastectomy rates were significantly increased in 2014 (62.4%) compared to 2004 (43.6%) [80].

Increased anxiety about disease recurrence and an overestimation of BC risk are related to higher rates of bilateral mastectomy [81,82]. On the other hand, the annual risk of contralateral BC is 0.25–1.25% and is further decreasing with the use of adjuvant treatment [83]. Conservative breast surgery is associated with increased 10-year survival compared to unilateral mastectomy and with no significant survival difference as far as the bilateral mastectomy is concerned [80]. The same result was published in the Asian setting by authors Sinnaduarai et al. [84]. Breast-conserving surgery followed by radiotherapy showed also better results in metastasis-free survival compared to mastectomy procedures [85]. Therefore, no survival benefit would justify the more aggressive approach in young women. There is also no evidence of worse outcomes in sentinel lymph node mapping in young patients, thus the indications are similar to the general population.

Furthermore, oncoplastic surgery techniques with immediate reconstruction should be offered to all patients undergoing breast-conserving surgery or mastectomy, except inflammatory BC, to maximize cosmetic effects and optimize self-image [73]. Young women with malignant disease should be managed by multidisciplinary specialized teams with an individualized approach to improve the age-specific diagnostic, therapeutic, and psychosocial issues typical for younger age including fertility preservation and family planning [73,86].

## 7. The Advanced Approach by Predictive, Preventive, and Personalized Medicine in Overall BC Management

### 7.1. Risk Assessment: Phenotyping and Genotyping 

The modern era is characterized by a BC pandemic. Moreover, the rising trend is even worse in young females below 40 including pregnant women. Therefore, it is necessary to reconsider current strategies in BC screening, diagnostics, and therapy. Importantly, BC occurring at younger age is particularly unpredictable with strongly promoted metastatic spread, lower survival rates, and worse response to adjuvant therapy. Also, the risk factors are not as clear as in the group of postmenopausal women [3]. Early BC risk assessment involves an assessment associated with genetic factors and an impact of environmental factors leading to cancer development.

Hereditary BC risk assessment focuses on the detection of mutations in dominant cancer-predisposing genes (*BRCA1*, *BRCA2*, or *p53),* which are responsible for approximately 20% of BC in the 30s age group. Furthermore, germline mutations in BC susceptibility genes, including *PTEN*, *STK11*, *CDH1*, *PALB2*, *ATM*, and *CHECK2,* have been connected with risk assessment in young premenopausal women, thus sequencing of these genes can provide valid data for cancer management [87,88,89]. Obviously, only few diseases, such as Cowden´s syndrome, exhibit typical phenotypes that are connected to the mutation in the *PTEN* gene. Therefore, sequencing analysis plays a crucial role in estimating the risk of hereditary BC in young women. Evaluation of the family history (age of BC onset, bilateral cancer, other types of early-onset tumor, etc.) demonstrates an important tool that analyzes the presence of predisposing genes in the family. There are several models of BC risk assessment including the Gail model, Claus model, BRCApro model or Tyrer-Cuzick model evaluating known risk factors [90,91].

Except for well-known risk factors, we recognize specific patient phenotypes that are often underestimated and act as a marker of disease predisposition and/or progression. Headaches with migraines are often seen as a paraneoplastic syndrome in patients with brain metastases. Moreover, migraines increase the risk of TNBC tumors. Additionally, increased ET-1 secretion in BC patients is also involved in the change of pain and temperature perception. Dehydration is also a strong risk factor for BC development. Altered thermoregulation caused by mitochondrial dysfunction induced by BC process is an important diagnostic and prognostic factor [5].

In the last years, an extensive focus is placed on chronic systemic hypoxia that is typical for Flammer syndrome (FS) on one hand, and higher risk of BC development on the other hand [30]. To this end, FS phenotype represents a new promising research area in cancer development and progression in general. FS typically appears at a younger age and is characterized by altered reaction of blood vessels and chronic hypoxia. This syndrome triggers molecular and microenvironmental changes with an increased risk of aggressive BC development even at a younger age [15,30,92]. The analysis of the questionnaire including 15 most relevant symptoms of FS (altered sense regulation, headaches, dizziness, psychological factors, thermoregulation etc.) showed an increased prevalence of studied symptoms in BC patients that confirms a crucial role of the cardiovascular component in BC pathology [30].

A series of publications presenting results of a multi-center study on the topic have been recently released demonstrating both—the Flammer syndrome (FS) phenotype, as well as the molecular pathways shifted in FS-individuals as being highly relevant for the BC development and progression [6,30,92,93,94,95]. Below the functional link between clearly described FS symptoms as appearing at early age [96] and risks known as strongly contributing to the development of metastatic breast cancer are summarized.

#### 7.1.1. Deficient Thermoregulation and Feeling Inappropriately Cold

Both phenomena are clearly described for the FS phenotype as functionally linked to the disturbed microcirculation [6]. On the other hand, those symptoms have been attributed to breast cancer being conducted by two mechanisms:

1. Insufficient energy production including systemic mitochondrial dysfunction and misguided DNA repair [97].
2. Systemic inflammatory processes [98,99,100] as reviewed elsewhere [5].

#### 7.1.2. Persistently Cold Extremities, Altered Endothelin-1 Blood Patterns, and Systemic Hypoxic Effects

Disturbed microcirculation results in cold extremities characteristic for individuals affected by the FS. On the other hand, accumulated evidence demonstrates that primary and secondary vascular dysregulation is a strong contributor to the cancer development and progression. At the molecular level overexpression of endothelin-1 is the biomarker for both—Flammer syndrome and breast cancer with particularly poor prognosis [101]. Increased endothelin-1 levels in blood cause inappropriate vasoconstriction, systemic hypoxic effects, and predisposition to aggressive metastatic disease [6,102,103].

#### 7.1.3. Reduced Thirst and Body Dehydration

FS-individuals are well-known for their altered sensitivity towards different stimuli: pain, thirst, smell, light, and stress provocation. If not controlled by mind, reduced thirst may lead to chronically decreased liquid intake and consequent body dehydration which is a strong risk factor for several pathologies including breast malignancies as well as headache/migraine attacks [104,105] typical for FS affected individuals as reviewed elsewhere [5].

#### 7.1.4. Altered Circadian and Sleep Patterns

Altered circadian and sleep patterns are characteristic for FS-phenotype [96]. Circadian and sleep patterns regulation is linked to immunomodulation, formation of the vasodilatator—nitric oxide, synthesis of melatonin and serotonin—all playing a key role in protection against breast cancer. Shifter circadian genes regulation is implicated in pathomechanisms of particularly aggressive metastatic breast cancer [106,107]. Extended information on a functional link between the Flammer syndrome phenotype and metastatic breast cancer is provided in the recently published book *Flammer Syndrome—From Phenotype to Associated Pathologies, Prediction, Prevention and Personalisation* [7].

**Figure 1:**
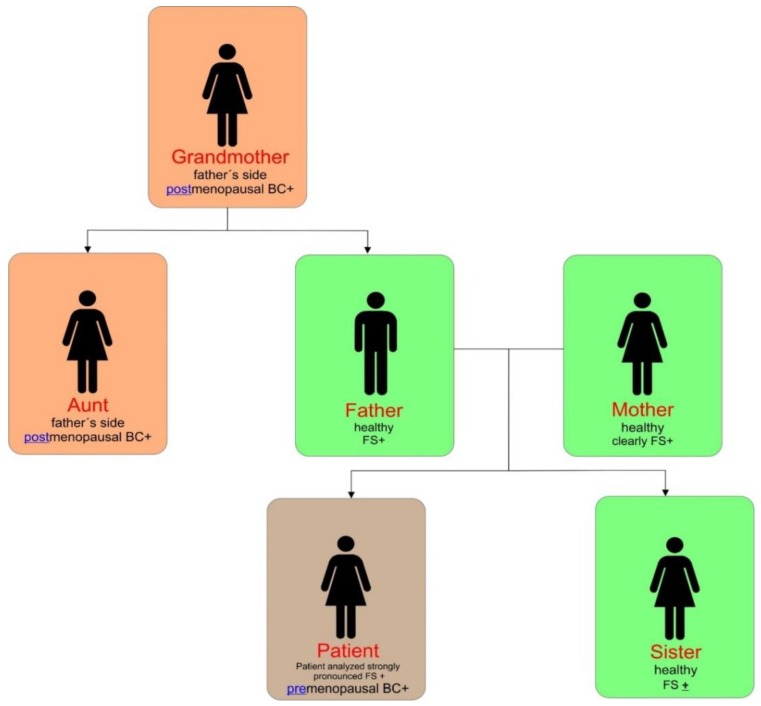
FS phenotype analyzed in the family members.

FS phenotype strongly contributes to BC development in young females. Accordingly, a TNBC case with pronounced FS phenotype has been analyzed utilizing the FS focused questionnaire [7]. The results are presented in Table 3. Moreover, Figure 1 shows a family tree analyzing FS phenotype and BC diagnosis.

After all, a reported patient was genetically predisposed to oncologic disease. However, the patient was the very first premenopausal BC case diagnosed at the age of 41 years. Further, no usual modifiable risk factors have been reported in the patient. On the contrary, it was the first time when the attention of caregivers was paid on specific symptoms and signs of FS in the family. The detection of most obvious symptoms and signs of FS was attributed to reported patient compared to other family members: clear vascular deregulation (inappropriate vasoconstriction), dizziness, altered thermoregulation (feeling cold in comparison with feeling of other people), low BMI, perfectionism, changes in sense regulation (absent feelings of thirst, strong perception of smell), and prolonged sleep onset [7]. Consequently, the doctor recommended the patient and affected family members to clarify lifestyle habits adapted to the needs of the FS phenotype. Further, the patient might undergo predictive diagnostics focused on enumeration and molecular analysis of circulating tumor cells in blood followed by the decision regarding chemo-preventive therapy.

### 7.2. Multi-Omic Diagnostic Approach

There is an urgent need for individualized multi-level diagnostics and risk assessment methods, including family history, questionnaires with phenotyping of individuals, imaging methods, and multi-omic approach [95]. Modern molecular technologies allow an extensive analysis of genes, transcripts, and proteins with the simultaneous quantitative and qualitative diagnostics [108]. The most recent development of DNA methylation, microRNA expression, and protein expression assays provide further possibilities for the evaluation of tumor malignant potential [109].

As mentioned previously, the frequency of the most aggressive forms of BC is higher in younger premenopausal women when compared to older patients. Therefore, it is necessary to find relevant markers suitable for prediction and risk evaluation of cancer. Moreover, a higher level of circulating resistin was associated with better prognosis of patients with early-onset BC. Analyzed data suggested that patients with higher expression rate of resistin represent tumor cases with less probability to metastasize [110]. In addition, the miRNA profile can be used as a screening tool for BC risk assessment in individual subjects. Moreover, it was found to possess better discriminatory power in younger women [77]. In summary, risk assessment of early-onset BC could be evaluated using numerous models or multi-omics approaches through the analysis of genome or proteome signature assessing prognosis and predicting patient outcomes.

Extensive breast proteomic assessment has demonstrated menopausal status-specific protein profiles to be involved in hormone and cytokine signaling pathways and regulation. Proteomic and metabolomic investigations, including plasma folate, vitamin B6, vitamin B12, and homocysteine, showed different BC risk, especially in premenopausal women. Moreover, subcellular imaging of chromosomal DNA revealed different Bcomet profiles in younger and older BC patients [3].

Liquid biopsy as a non-invasive universal approach for sampling the genomic and epigenomic signatures, such as circulating tumor DNA (ctDNA), circulating tumor cells (CTC), lncRNA, mRNAs, miRNAs, proteins, and exomes, should be the crucial part of diagnostics of premalignant disease as well as early-stage tumors and could serve as a powerful prognostic and predictive tool. Liquid biopsy is characterized by both high sensitivity and specificity with real-time dynamic monitoring [111].

Human milk also offers the opportunity to find potential BC biomarkers of women in reproductive age. The collection of milk is non-invasive and it is possible to obtain bilateral samples. Breast milk contains epithelial and immune cells together with secreted proteins from the targeted tissue. Aslebagh et al. [112] showed that xanthine dehydrogenase/oxidase and lipases were downregulated in BC patients, whereas the upregulation was associated with alpha-amylase, gelsolin, alpha-2- glycoprotein 1, apoptosis-inducing factor 2, and vitronectin [112].

An analysis of proteins in the tear fluid is another non-invasive method. A modified expression of several proteins associated with the immune system (C1Q1, S100A8) and metabolic cascades (ALDH3A or TPI) [113] have been identified in BC patients. The selected molecular patterns from tear fluid could differentiate malignancies from benign lesions and healthy women with specificity and sensitivity attacking 70% [114].

### 7.3. BC Prediction, Machine Learning, and Artificial Intelligence

Management of BC depends on the multi-professional cooperation, stratification of patient risk, improvement in the understanding of molecular mechanism and pathology, as well as prognostic and predictive modeling. Nevertheless, due to the heterogeneity, an assignment to mentioned subgroups is not adequate for the establishment of the newest strategies of clinical management [52].

The multi-omic approach is able to distinguish between TNBC, other BC subtypes, and disease-free controls and could be used for specific TNBC group of young women. Golubnitschaja et al. [95] demonstrated a consolidated increase in preTNBC as well as postTNBC homocysteine levels. Importantly, particularities in thiol-regulation and redox control specifically attributed to TNBC subtypes are required to be reflected in the overall stress response [95]. The great predictive power of multi-level diagnostics was related to an innovative hybrid-omic approach with activities of MMP-9 in blood serum as well as the rate of RhoA expression in peripheral leukocytes to be applied in prediction of tumor progression and BC patients stratification [93]. As was demonstrated by Frőhlich et al. [3], the usage of premenopausal BC-specific multi-omic signature may be applied for the stratification of patients with benign breast changes. Moreover, it can be also relevant for high confidence (>90%) determination between high and low BC-risk. The multi-omic signature includes oxidative stress factors, homocysteine, and filamentous actin due to the key role in the carcinogenic process [3].

Other prognostic and predictive markers are represented by specific lncRNAs (long non-coding RNAs) that correlate with mammary carcinogenesis. New biomarkers for efficient individualized therapy such as lncRNAs (SNGH 12, HIF1A-AS2) are necessary. Many of them could play an important role in endocrine regulatory resistance, e.g., tamoxifen resistance [115].

BC survivability prediction represents a challenging task that could strongly benefit from the development of personalized predictive models [116]. Prognostic and predictive models are tightly connected to the rapid development of digital technologies that could help to analyze numerous data of personalized medicine. Recently, an introduction of machine learning (ML) led to the development of models of prognostic classification that can be used to predict the outcome of individual cancer patients. In the study analyzing major nonlinear machine learning methods, ER/PR/HER2 and breast surgery status strongly influenced survival while the gene expression cluster was a moderately influential factor [117]. Precise oncology beyond well-known pattern recognition represents a significant potential attributed to ML. Reduction of the number of false-positive cases, decrease in the time of patient´s examination, as well as benefits in the interpretation and screening as a second messenger are considered as advantages of the usage of BC diagnosis developed systems [118].

The most important advantage of artificial intelligence and the deep learning approach is the analysis of multi-omics data influencing the carcinogenic process of BC. There is a connection between genotypes and phenotypes expressed in genomic and phenomic data mediated via the conversion of molecular-scale genotype information into a macroscale presentation of a specific phenotype of an organism. The identification of transformation drivers is considered to be the gold mine of individualized therapy. An importance of a deep learning approach depends on the capability to find new information concerning regulatory processes and biological systems as well as the determination of causal variants of the disease [119].

## 8. Conclusions and Future Directions

Despite that postmenopausal women represent the majority of BC patients, an age-profiling of BC is currently shifting as a response to the growth of the cohort of young premenopausal BC patients [1,2]. The heterogeneous nature of the genotype and phenotype of premenopausal BC [3], often asymptomatic course of the disease [5], insufficient screening programs for younger women [8], complications in PABC diagnostics [12,13], along with more aggressive subtypes of BC in young women [4,57] contribute to an increase in early-onset BC. Therefore, an urgency in the novel alternations of diagnostic, prediction, prognosis, and therapy approaches is a necessary basis of new strategies of clinical management focused on young premenopausal females [45]. Recent evidence suggests the possibility of using a combination of molecular signatures including ctDNA, CTC, lncRNA, mRNAs, miRNAs, proteins, and exomes as high perspective markers of liquid biopsy that will contribute to the better therapy intervention as a part of personalized medicine in the near future [110]. Panels of previously mentioned molecular markers, as well as an enormous progress in gene expression analysis and next gene sequencing, represent a perspective area of identification of patients with high risk of early-onset BC and they also predict prognosis and appropriate therapeutic approaches [77,120]. Novel insights in the field of BC origin in young women are associated with a particular interest in FS that generally affects groups of premenopausal women with a high risk of cancer initiation at a young age for which an increased prevalence of the aggressive phenotype is typical. There is increasing evidence that relevant FS symptoms occur in a higher incidence in BC patients. Therefore, the frequently analyzed FS symptoms can identify individuals with high-risk of early-onset BC and can also help to stratify patients in accordance with the assessment of the possibility of cancer development [8]. Noteworthy, FS phenotype might be relevant also for other cancer types in women as well as in men. This concept has been recently presented by a series of follow up studies analyzing the symptoms of vaginal dryness and “dry mouth” syndrome [121,122]. However, more complementary research is needed to demonstrate the impacts of FS phenotype for other cancers.

Currently, public health is facing a major challenge represented by an individual approach suitable for a cohort of young-premenopausal BC patients regarding differences in the prevalence of molecular subtypes and selection of an appropriate therapy. Novelties such as serum/plasma miRNA panels or targeted DNA panels evaluating differences in expression levels and mutation rates combined with symptoms and molecular signatures of FS and innovations in the field of screening and diagnostic technologies represent a potent perspective in the management of BC occurring in a cohort of very young women [8,15,123]. In conclusion, different approach in BC prediction, screening, diagnostics, and targeted therapy could lead to a substantial drop in BC cases in the female population.

## Figures and Tables

**Table 1:** Modifiable and non-modifiable BC risk factors.

MODIFIABLE Risk Factors	NON-MODIFIABLE Risk Factors
Body mass index	BRCA1, BRCA2 mutations
Parity	Li Fraumeni syndrome (p53)
High alcohol intake	CHEK2*1100delC mutations and other genetic alterations
Smoking	Age
Lifestyle	
Breastfeeding	
Radiation exposure in utero	

**Table 2:** Molecular signatures of early-onset breast cancer.

Molecular Signatures Groups	Most Common Genetic Alterations
Genomic alterations	SEPP1, ESR1, IL1RN, SCD, TIAM1, UBE2C, CCNB2, CEP55, TOP2A, BIRC5, TPX2, SHCBP1, KIAA0101, PTTG1, UBE2T, DEPDC1, NUSAP1, CCNB1, HELLS, KIF4A, RRM2, IGF1R, APOBEC3A/B, amplification of 11q13 (CCND1), 17q12 (ERBB2), Chr1p34, and copy number loss at Chr15q13
Inflammatory biomarkers	TNF-308G>A polymorphism
miRNA	miR-1228, miR-3196, miR-1275 miR-1207, miR-92b, miR-139, miR-183, miR-182 and miR-96, miR-320, miR-10a, miR-130, miR-127-3p, miR-143, miR-10b, miR-125b, and miR-195
Signaling pathways	RANK/RANKL

**Table 3:** FS-phenotype specific signs and symptoms strongly pronounced in the patient.

Questions	Answers (Yes/No)	Comments
Cold hands and/or feet	Yes	Very frequently
Feel cold	Yes	Very soon
Low blood pressure?	Yes	Very frequent
Dizziness	Yes	Very frequent
Prolong sleep onset	Yes	Very frequent
Do not feel thirsty	Yes	Even in hot weather
Headache/Migraine	No	
Accompanying symptoms (e.g., visual disturbances)	No	
Altered reaction towards drugs	Not known	
Altered pain sensitivity	No	
Strong smell perception	Yes	Extraordinary pronounced
Slim at 20–30 years of age	Yes	Extraordinary pronounced
Tendency towards perfectionism	Yes	Strongly pronounced
Tinnitus	No	
Reversible blotches (white or red) on your skin e.g., in stress situations	Yes	Strongly pronounced
